# Treatment Outcomes of Patients with Glomus Tympanicum Tumors Presenting with Pulsatile Tinnitus

**DOI:** 10.3390/jcm10112348

**Published:** 2021-05-27

**Authors:** Seung-Jae Lee, Sang-Yeon Lee, Gwang-Seok An, Kyogu Lee, Byung-Yoon Choi, Ja-Won Koo, Jae-Jin Song

**Affiliations:** 1 Department of Otorhinolaryngology-Head and Neck Surgery, Seoul National University Bundang Hospital, 300 Gumi-dong, Bundang-gu, Seongnam 13620, Korea; log8282@hanmail.net (S.-J.L.); choiby2010@gmail.com (B.-Y.C.); jawonkoo@snubh.org (J.-W.K.); 2 Department of Otorhinolaryngology-Head and Neck Surgery, Seoul National University Hospital, 101 Daehak-ro, Jongno-gu, Seoul 03080, Korea; maru4843@hanmail.net; 3 Music and Audio Research Group, Graduate School of Convergence Science and Technology, Seoul National University, Gwanak-gu, Seoul 08826, Korea; angoang2@naver.com (G.-S.A.); kyogu.lee@gmail.com (K.L.)

**Keywords:** tinnitus, pulsatile tinnitus, glomus tympanicum, paraganglioma

## Abstract

We reviewed the clinical characteristics and treatment outcomes of patients with glomus tympanicum tumors (GTTs) presenting with pulsatile tinnitus (PT). We explored whether transcanal sound recording-spectro-temporal analysis (TSR-STA) usefully evaluated changes in PT. The medical records of 13 patients who underwent surgical removal of GTTs were reviewed retrospectively. Two patients underwent preoperative endovascular embolization. Changes in PT, pre- and postoperative audiometry data, TSR-STA results, and clinical outcomes were evaluated. PT was the chief complaint in eight patients (61.5%) and resolved immediately after surgical intervention in all. Two patients exhibited ipsilateral, pseudo-low-frequency hearing loss (PLFHL); surgical GTT removal elicited postoperative improvements in the ipsilesional low-frequency hearing thresholds. Five patients underwent TSR-STA using previously described methods. TSR-STA revealed definite rise-and-fall patterns; surgical tumor removal abated this pattern in one patient, but, for the other four, the patterns did not change greatly post-intervention. Thus, GTT-related PT can be treated successfully (via surgical GTT removal) without complications. In selected cases, preoperative embolization reduces intraoperative hemorrhage. In PT patients with PLFHL, a detailed otoendoscopic examination of the middle ear is required to rule out a GTT. TSR-STA may usefully (and objectively) assess postoperative improvements in GTT-related PT.

## 1. Introduction

Paragangliomas are a subset of neuroendocrine tumors with very rich vascular networks; the tumors originate embryologically from the paraganglionic chief cells of the autonomic nervous system [1,2]. A glomus tympanicum tumor (GTT), also termed a tympanic paraganglioma, is one of the most common benign tumors of the head and neck, especially in the mesotympanum of the middle ear cavity. A GTT most frequently arises from a glomus chemoreceptor station that lies along the tympanic plexus of Jacobson’s nerve (IX) or along the auricular branch of Arnold’s nerve (X) [3]. Although GTTs are usually pathologically benign and only infrequently induce autonomic dysfunction caused by catecholamine release, persistent growth can be problematic given the proximity of the tumors to the cochlea, jugular vein, cranial nerves, and carotid artery [4].

A GTT typically presents as a reddish mass under the tympanic membrane and is commonly associated with pulsatile tinnitus (PT), given its highly vascular nature and typical anatomical position [2]. Depending on the tumor size and extent of tumor adherence to surrounding structures, conductive or sensorineural hearing loss and vertigo may develop at later stages of tumor growth [5]. On the other hand, small GTTs may be asymptomatic and are usually found incidentally.

To date, complete surgical resection is the definitive treatment for GTTs. Surgery is associated with a high rate of tumor control and elimination of aural symptoms, including PT, without significant complications [4]. Although surgical removal is generally satisfactory, the highly vascular nature of the tumor, the small volume of the middle ear space, and tumor extension to surrounding cranial structures may render complete surgical resection challenging [6,7] and associated with residual symptoms. Furthermore, the lack of any objective assessment of treatment outcomes renders it difficult to ascertain the efficacy of GTT removal. Objective tests are needed to identify if a GTT is the cause of PT and to confirm that surgical intervention really improves PT symptoms.

In this perspective, the authors have reported a technique called “transcanal sound recording and spectro-temporal analysis (TSR-STA)” to objectively evaluate and differentially diagnose the PT in various ways. Specifically, TSR-STA can visualize heartbeat-synchronous patterns, peak, and RMS amplitudes of sound pressure waves of PT pre- and postoperatively, which in turn may usefully quantitate preoperative symptoms and evaluate postoperative symptom improvements in a range of vascular PT. In this study, we examined the clinical characteristics and treatment outcomes of patients with GTTs presenting with PT and analyzed whether TSR-STA usefully evaluates improvements in PT.

## 2. Materials and Methods

### 2.1. Subjects

We retrospectively reviewed the medical records of 13 patients who underwent surgical removal of GTTs between April 2003 and July 2020 at Seoul National University Bundang Hospital. The study protocol, and a waiver of any need for informed patient consent (given the retrospective nature of the chart review), were approved by the review board of the Clinical Research Institute of Seoul National University Bundang Hospital (IRB no. B-2104-678-102). All procedures adhered to approved institutional guidelines and those of the Declaration of Helsinki.

### 2.2. Otoendoscopic and Radiological Evaluations

**Figure 1:**
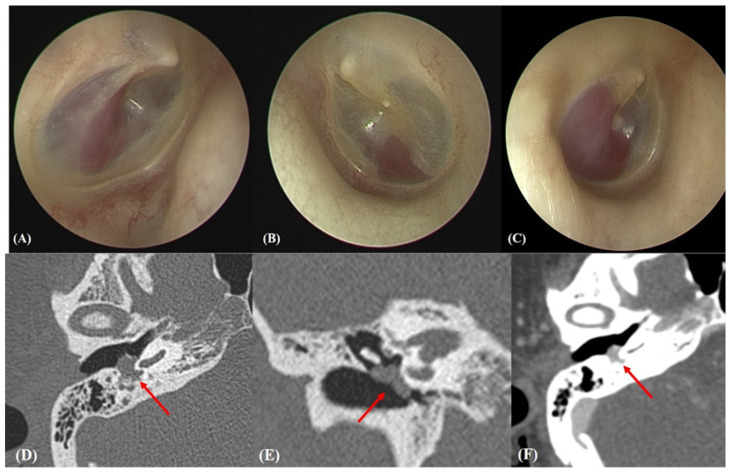
(**A**–**C**) Preoperative endoscopic views of subjects 4, 5, and 6, respectively. In each case, a reddish round mass abuts the tympanic membrane. (**D**–**F**) Preoperative temporal bone computed tomography (TB-CT) images of subject #6. The initial axial (**D**) and coronal (**E**) TB-CT images (without contrast enhancement) reveal an 8 mm-sized mass (arrows) in the right middle ear lateral to the cochlear promontory that abuts the long process of the incus, stapes, and tympanic membrane. (**F**) The initial axial TB-CT image (with contrast enhancement) reveals a middle ear mass suggestive of a glomus tympanicum tumor (arrow).

GTT tumors were confirmed otoendoscopically (Figure 1A–C) and via contrast-enhanced computed tomographic angiography of the temporal bone (TB-CTA) using a Philips 128 CT scanner (Philips Medical Systems). TB-CTA images were obtained at a 0.4 mm slice thickness, 120 kV, collimation of 64 × 0.6 mm, and 205 mAs; over a 1-s rotation; using a pitch factor of 0.85. All parameters were appropriate given the ages of the subjects. A GTT was defined as a single, round solid tumor confined to the mesotympanum, with highly vascular portions evident in contrast-enhancement images (Figure 1D–F).

### 2.3. Subjective Evaluation

All patients underwent audiological evaluation, including pure tone audiometry (PTA) pre- and postoperatively. Pre- and postoperative PTA thresholds were determined for each patient’s right and left ear at the frequencies of 0.5, 1, 2, and 4 kHz. As previously described, pseudo-low-frequency hearing loss (pseudo-LFHL) (i.e., an ipsilateral hearing threshold greater than 10 dB HL at both 250 and 500 Hz, or greater than 20 dB HL at either 250 or 500 Hz relative to the contralateral side) in the ipsilesional ear and improvement thereof after surgery were assessed [8].

In five patients (subjects 4, 5, 6, 7, and 8), subjective tinnitus loudness was evaluated using a numeric rating scale (NRS, answering the question “how loud is your tinnitus?” on a scale from 0 to 10). In the other eight patients, subjective symptom changes were not so evaluated.

### 2.4. Objective Evaluation

TSR-STA was performed to objectively confirm GTT-induced PT and analyze the psychoacoustic characteristics thereof using previously described methods [9,10,11]. Transcanal sound recording was performed with patients in several positions, including a neutral sitting position, a supine position with and without head rotation to the ipsilateral and contralateral sides. All data were collected with patients in a soundproof audiometric booth. An external auditory canal (EAC)-sealing omnidirectional condenser lavalier microphone (RODE Micro-phones, Sydney, Australia) was tightly inserted to record the sound pressure wave produced by the GTT. A lavalier microphone with sensitivity up to −33.5 dB re 1 V/Pa (21.00 mV at 94 dB SPL) was used to record the sounds. The signals were recorded using Cubase 5.0 software (Steinberg Media Technologies GmbH; Hamburg, Germany) at a sampling rate of 44,100 Hz and analyzed with the aid of MATLAB ver. 2020b (MathWorks, MA, USA). For temporal analysis, the recorded signals were low-pass filtered to 15 Hz using a tenth-order Butterworth filter to analyze periodic heartbeat synchronous patterns of sound waves. STA featured a brief Fourier transform over the frequency range of 20–8000 Hz, and a Hanning window of ~50 ms (2048 samples) was used with a hop size of ~10 ms (512 samples). The magnitude scale was converted to dB SPL [9].

TSR-STA and changes in pseudo-LFHL are the only two methods other than questionnaires to methods to evaluate the changes in PT after surgical- or interventional procedures. However, as changes in pseudo-LFHL can inherently be affected by the patients’ compliance, TSR-STA has the advantage as the only objective measure for evaluating changes in PT, although it is not commercially available, and it needs several steps of signal analysis.

### 2.5. Surgical Management

All patients underwent surgical GTT removal under general anesthesia via the trans-canal or retro-auricular approach, depending on the extent and location of the tumor. Before surgical treatment, a subset of patients underwent transfemoral cerebral angiography (TFCA) and embolization of vessels feeding the tumor; this minimized intraoperative hemorrhage. All specimens were sent to the Department of Pathology for pathological confirmation.

### 2.6. Statistical Analysis

Descriptive statistics were used to describe the demographics and clinical characteristics of patients with GTTs. Data are presented as the means ± standard deviations. Continuous variables were subjected to normality testing. The differences between pre- and post-treatment hearing thresholds and NRS loudness scores were compared using the Wilcoxon signed-rank test. All data were analyzed with the aid of Statistical Package for the Social Sciences software for Windows (version 20.0; SPSS, Inc., Chicago, IL, USA). Statistical significance was set at *p* < 0.05.

## 3. Results

### 3.1. Demographic and Clinical Characteristics

The demographic and clinical characteristics of all 13 patients are summarized in Table 1. All were female and of average age 56.62 ± 16.56 years (range, 34–80 years). Ten presented with right-sided GTT. As summarized in Table 1, PT was the most common chief complaint (61.5%, 8/13), followed by otalgia (15.4%, 2/13). The remaining three patients (23.1%) were incidentally diagnosed on otoscopic examination (they lacked symptoms). The average symptom duration was 4.54 ± 4.18 months.

**Figure 2:**
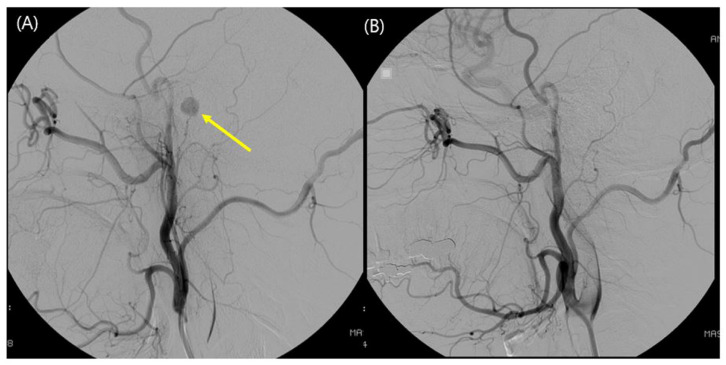
Preoperative transfemoral cerebral angiography (TFCA) and embolization in subject 7. (**A**) A highly vascular 6 mm-sized oval nodular lesion (yellow arrow) fed by the inferior tympanic artery from the ascending pharyngeal artery was observed during TFCA; it was suggestive of a glomus tympanicum tumor. (**B**) After superselective angiography and confirmation of the absence of any dangerous anastomosis, the feeding vessel was embolized without any complications, and the mass became invisible.

Six patients underwent preoperative TFCA to evaluate the vessels feeding the tumor. Selective embolization was performed to block collateral feeding vessels from the vertebral artery and ascending pharyngeal artery in subjects 5 and 7, respectively (Figure 2). Embolization could not be performed in the other four patients because the feeding vessels were narrow in diameter, and the risk of potential complications outweighed the advantages. No serious complications developed during or after the intervention.

### 3.2. Intraoperative Findings

**Figure 3:**
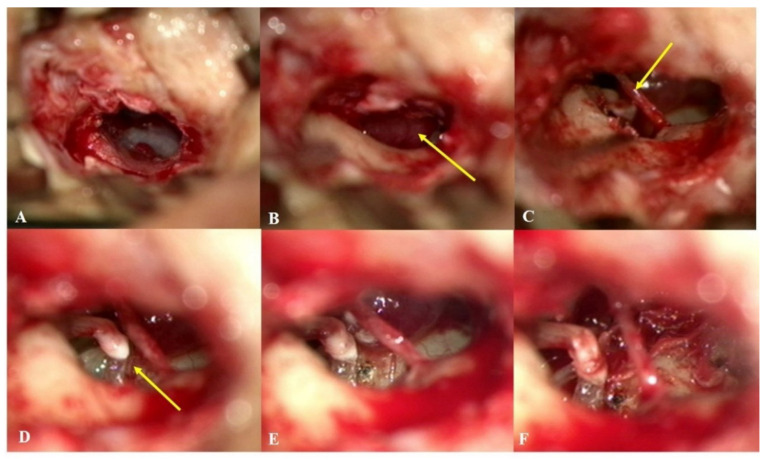
Intraoperative microscopic images of subject 8. (**A**) An endaural approach was chosen in this case. (**B**) After the elevation of a tympanomeatal flap, a round, reddish tumor (arrow) was observed in the right mesotympanum. (**C**) The chorda tympani nerve (arrow) was mobilized to improve the exposure of the mass. (**D**) The incudostapedial (I-S) joint (arrow) was located and separated to protect the stapes while mobilizing the mass abutting the incus. (**E**) A 6 mm-sized glomus tympanicum tumor with a stalk arising from the promontory was observed. (**F**) After the feeding vessel was cauterized with a CO2 laser, the tumor was fully removed en-bloc without any injuries to critical structures or severe hemorrhage. The I-S joint was immediately reconnected using bone cement.

As summarized in Table 2, of the 13 patients, eight (61.5%) underwent surgery via the endaural approach, three (23.1%) via a combined retroauricular and transcanal approach, one (7.7%) via a transcanal approach, and one (7.7%) via a retroauricular approach. In all cases, the GTTs were localized in the mesotympanum and were in contact with the tympanic membrane. In all cases, GTTs were dissected using cold instruments and bipolar cauterization; a CO2 laser was used in selective cases to debulk the lesion or cauterize a feeding vessel (Figure 3). Four patients (subjects 3, 6, 9, and 12) underwent tympanoplasty; tragal perichondrium was used to restore tympanic membrane defects that developed during tumor removal. All lesions were completely removed without any immediate postoperative complications. The final pathological reports confirmed that all tumors were highly vascular GTTs.

### 3.3. Subjective Outcomes

**Figure 4:**
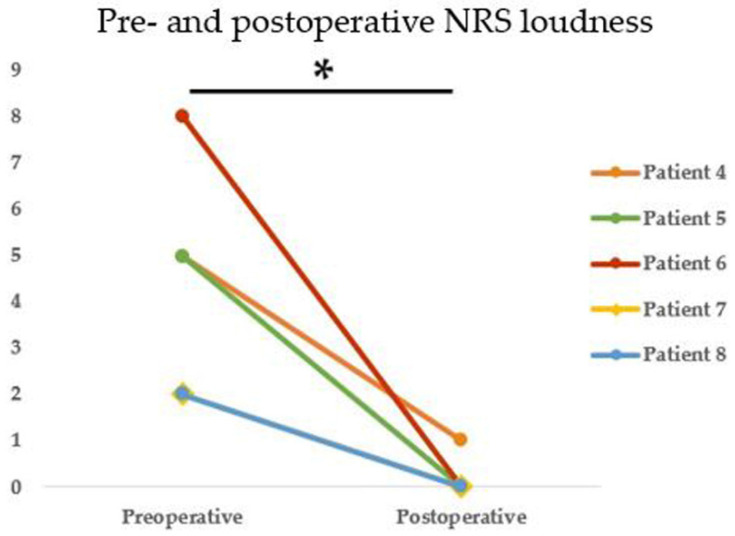
Pre- and postoperative NRS loudness scores indicated significant, marked improvements after surgery in all five patients (subjects 4, 5, 6, 7, and 8) (*p* = 0.042, * Indicates statistical significance by the Wilcoxon signed-ranks test).

PT abated immediately after surgical intervention in all eight patients who presented with PT. During the follow-up period (19.62 ± 21.57 months), no tumor recurrence or aural symptom, including PT, occurred. As shown in Table 1, the subjective (NRS) loudness score decreased markedly after surgery in all five patients (subjects 4, 5, 6, 7, and 8); the mean preoperative NRS score was 4.4 ± 2.4, and that postoperatively was 0.2 ± 0.4; the difference was significant (*p* = 0.042, Wilcoxon signed-rank test) (Figure 4). Although NRS loudness was not checked in the other eight subjects, all also reported complete PT resolution postoperatively.

**Figure 5:**
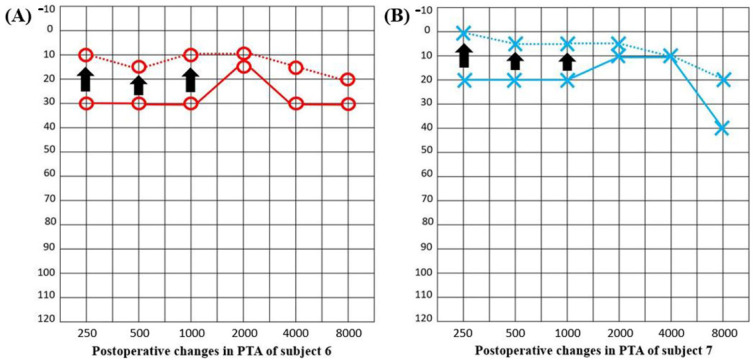
(**A**) Postoperative evaluation of the pure tone averages of subject 6 revealed improved hearing thresholds (by 20 dB at 250 Hz and 15 dB at 500 Hz) (arrows). (**B**) Postoperative evaluation of the pure tone average of subject 7 also revealed improved hearing thresholds (by 20 dB at 250 Hz and 15 dB at 500 Hz) (arrows).

All but four patients (subjects 3, 6, 7, and 11) exhibited normal hearing thresholds across all frequencies preoperatively. Subjects 3, 6, 7, and 11 evidenced mild-to-moderate sensorineural hearing loss without a conductive component. The preoperative pure tone average (20.71 ± 13.51 dB) did not differ significantly from the postoperative pure tone average (21.92 ± 15.31 dB) (*p* = 0.223, Wilcoxon signed-rank test). Of the 13 patients, two (15.4%) presented with preoperative pseudo-LFHL. Notably, their audiograms revealed markedly decreased thresholds at low frequencies (0.25 and 0.5 kHz). However, the pseudo-LFHL resolved in both subjects (Figure 5). Both exhibited improvements of 20 dB and 15 dB at 0.25 and 0.5 kHz, respectively.

### 3.4. Objective Outcomes

**Figure 6:**
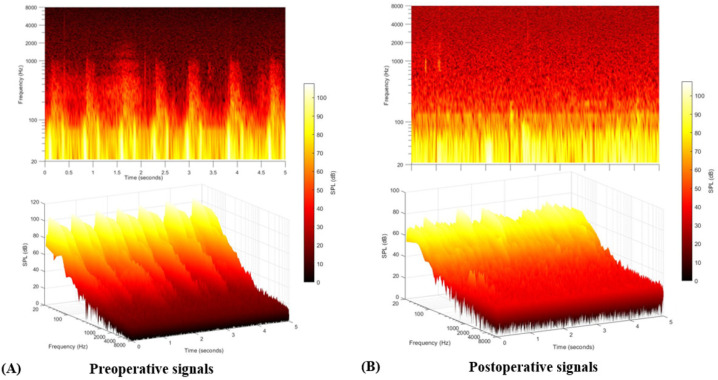
(**A**) Pre- and (**B**) postoperative two-dimensional spectrograms and three-dimensional waterfall diagrams of subject 11. Pulsatile, synchronized signal patterns are objectively evident in the preoperative spectrogram and waterfall diagram; these disappeared after tumor removal.

TSR-STA was performed to objectively confirm PT and analyze the psychoacoustic characteristics of the PT, using previously described methods. Of the 13 patients, five underwent both pre- and postoperative TSR-STA. Preoperatively, a definite rise-and-fall pattern synchronized with the heartbeat was observed in subject 11. In this subject, both the peak and RMS amplitudes became more significant, especially when the head was rotated to the contralateral side. After GTT removal, the pulse-synchronous signals abated markedly (Figure 6). For the other four patients, pre- and postoperative TSR-STA failed to reveal any typical PT-related signals, probably due to technical problems in the recording system rather than the nature of the PT.

### 3.5. Complications

No immediate postoperative complications were observed. No long-term severe morbidity or mortality was reported over an average follow-up period of 19 months (range, 12–54 months) after surgery, except for delayed grade II facial nerve paralysis (FNP) in subject 2 at 2 weeks postoperatively. This was resolved completely within 1 week without any further complications.

## 4. Discussion

We explored the clinical characteristics and treatment outcomes of patients with GTTs presenting with PT. GTT-induced PT can be successfully treated surgically; in selected cases, preoperative embolization reduces intraoperative hemorrhage. PT symptom improvement was demonstrated subjectively (NRS loudness scores) and objectively (changes in the TSR-STA outcome and pseudo-LFHL improvement). Our results build on previous studies on the management of middle ear paragangliomas and provide clinical guidelines for the management of GTT-induced PT with objective PT testing.

Recording the patient’s history and a physical examination is imperative when evaluating a patient with a suspected GTT. Radiological examinations are required to detect underlying vascular causes such as an aberrant internal carotid artery or a glomus jugulare tumor; either can co-exist with a GTT [12,13]. Several authors have reported that GTT patients commonly present with a complaint of PT [2,4], as we also found. Although all of our patients exhibited reddish, pulsatile round tumors in contact with the tympanic membrane, some (23.1%, 3/13) reported no definite aural symptoms (PT, subjective hearing loss, aural fullness, or otalgia). We believe that small GTTs are asymptomatic and usually found incidentally [14]. Hearing loss per se may hinder the perception of PT generated by the GTT vasculature and then directly transmitted to the inner ear or tympanic membrane [15,16,17]. Although GTTs are not associated with malignant transformation or metastasis, persistent growth and local tissue destruction can trigger devastating disability if the GTT is not treated in a timely manner [18]. Surgery is mandatory and should not be delayed even if a tumor is incidentally detected and the patient lacks symptoms.

We found that GTT-induced PT could be successfully treated via surgical tumor removal. No patient exhibited any aggravation of PT over a follow-up period of up to 54 months. Complete surgical resection is the only cure for a GTT [2,4], as it ensures a high rate of tumor control and resolution of aural symptoms with a low risk of complications. A high success rate such as ours may be attributable to our tailoring of the surgical approach according to the extent and location of the GTT. Preoperative embolization attenuates intraoperative hemorrhage and reduces tumor size [19]. Although any role for such embolization remains a topic of debate, especially in patients with tympanic paragangliomas, the reductions in tumor size and intraoperative hemorrhage enhance tumor visualization and manipulation of the surrounding structures, in turn facilitating functional preservation [20]. Preoperative embolization seeks to occlude vessels that feed the tumor, and the risk of complications is generally low [19,20]. We found that, in selected cases, preoperative embolization reduced intraoperative hemorrhage and thus sustained PT improvement after surgery.

Surgical GTT removal affords favorable outcomes in most cases; however, objective methods with which to evaluate symptom changes are needed. Objective tests for PT, including TSR-STA, usefully evaluate GTT-induced PT changes after surgery. Furthermore, the roles played by objective tests are unlikely to wane, given the importance of comparing findings among centers. In our current study, TSR-STA was used to evaluate selected patients pre- and postoperatively. Although PT changes were not apparent in some cases, probably because of a problem intrinsic to the recording system, the objective signal analysis revealed that peak and RMS amplitudes decreased after surgery, along with subjective PT improvement, in some patients. Moreover, the pseudo-LFHL that had masked the pulsatile sounds produced by the highly vascular tumors almost disappeared after surgery, indirectly confirming that GTT removal was successful. Thus, in PT subjects with pseudo-LFHL, a detailed otoendoscopic examination of the middle ear is required to rule out a GTT. Furthermore, TSR-STA may usefully quantitate preoperative symptoms and evaluate postoperative improvements in some subjects with PT caused by a GTT.

Our data complement those of previous studies on GTT management in terms of tumor control and PT resolution and can be used to establish valuable clinical guidelines for the management of GTT-induced PT. However, our work had certain limitations. Most importantly, our sample size was small. Additionally, any retrospective study is at risk of selection bias. In terms of tinnitus severity, only NRS loudness scores were available for some patients (given the retrospective nature of the study); moreover, such data are subjective. The addition of subjective questionnaires such as the THI and TQ, as well as other NRS scoring systems, would improve the quality of the subjective data. Finally, TSR-STA was performed only in 5 of 13 subjects included in the current study, and it revealed noticeable improvement in only one patient; the other four exhibited slight improvements (much less than the symptom improvements). This is because only five subjects who were operated on by one surgeon (J-J.S.) underwent TSR-STA, and there might have been a technical problem in the microphone considering that previous subjects who had abnormal vascular structures in the middle ear (i.e., an aberrant carotid artery, high jugular bulb, or GTT) contracting the tympanic membrane showed stark rise-and-fall patterns on preoperative TSR-STA [9,10]. In this regard, more data should be collected using a different device. Future prospective studies with more patients featuring long-term follow-up and more accurate examination methods are required to better understand GTT-induced PT and demonstrate the clinical utility of objective PT testing.

## 5. Conclusions

GTT-induced PT can be successfully treated via surgery. In selected cases, preoperative embolization reduces intraoperative hemorrhage. In patients with PT and pseudo-LFHL, a detailed otoendoscopic examination of the middle ear should be performed to rule out a GTT. Although we here observed changes in TSR-STA in one subject, follow-up studies with more cases are warranted to further validate TSR-STA as an objective tool to assess changes in PT after surgical removal of GTT, as we could not demonstrate such changes in the other four cases probably due to technical issues in the measuring system.

## Figures and Tables

**Table 1:** Demographic and clinical data for all 13 patients with glomus tympanicum tumors.

Pt	Sex	Age (Year)	Site	Symptom Duration (Months)	Follow-Up Period (Months)	C.C	Preop PTA (dB)	Postop PTA (dB)	Preop NRS Score for Loudness	Postop NRS Score for Loudness
1	F	39	R	3	49	PT	10.84	10	N/A	N/A
2	F	80	L	1	15	PT	29.17	32.5	N/A	N/A
3	F	80	R	1	7	PT	43.33	48.33	N/A	N/A
4	F	56	R	6	6	PT	9.17	12.5	5	1
5	F	68	L	12	5	PT	21.67	32.5	5	0
6	F	45	R	3	5	PT	22.5 (LFHL)	10.83	8	0
7	F	34	L	11	38	PT	13.33 (LFHL)	5	2	0
8	F	63	R	7	2	PT	24.17	29.17	2	0
9	F	52	R	0	15	Incidentally	7.5	10	N/A	N/A
10	F	38	R	0	26	Incidentally	3.33	6.67	N/A	N/A
11	F	77	R	0	2	Incidentally	42.5	42.5	N/A	N/A
12	F	41	R	5	73	Otalgia	7.5	8.33	N/A	N/A
13	F	63	R	10	12	Otalgia	34.17	36.67	N/A	N/A

Abbreviations: Pt, Patient; PT, Pulsatile tinnitus; PTA, Pure tone audiometry; C.C, Chief complaint; LFHL, Low-frequency hearing loss; NRS, Numeric rating scale; N/A, Not available.

**Table 2:** Demographic data and a summary of the surgical features.

Pt	Surgery	Preop. TFCA	Preop. Embolization	Feeding Vessel	Postop. Complications	Final Pathology
1	Transcanal	N	N	Unknown	None	GTT
2	Retroauricular transcanal	N	N	Unknown	None	GTT
3	Endaural, T1	Y	N	Distal small branch of the APA	None	GTT
4	Retroauricular	Y	N	Small petrous branch of the MMA	None	GTT
5	Endaural	Y	Y	Collateral vessels of the VA	None	GTT
6	Endaural, T1	Y	N	Collateral vessels of the APA, IMA	Delayed FNP	GTT
7	Retroauricular transcanal	Y	Y	Collateral vessels of the APA	None	GTT
8	Endaural	Y	N	Small branch of the ECA	None	GTT
9	Endaural, T1	N	N	Unknown	None	GTT
10	Retroauricular transcanal	N	N	Unknown	None	GTT
11	Endaural	N	N	Unknown	None	GTT
12	Endaural, T1	N	N	Unknown	None	GTT
13	Endaural	N	N	Unknown	None	GTT

Pt, Patient; Y, Yes; N, No; TFCA, Transfemoral cerebral angiography; T1, Tympanoplasty type 1; APA, Ascending pharyngeal artery; MMA, Middle meningeal artery; VA, Vertebral artery; IMA, Internal maxillary artery; ECA, External carotid artery; FNP, Facial nerve paralysis; GTT, Glomus tympanicum tumor.

## Data Availability

Data sharing not applicable.
